# Influence of competitive attitude and self-efficacy on task motivation in vocational high school students: the moderating role of competitive environment in the context of ‘Lying Flat’ culture

**DOI:** 10.3389/fpsyg.2024.1427041

**Published:** 2024-10-17

**Authors:** Cheng Ma, Bo-Ching Chen

**Affiliations:** ^1^Department of Physical Education, University of Shanghai for Science and Technology, Shanghai, China; ^2^Undergraduate Program of Sports Coaching, CTBC Business School, Tainan, Taiwan

**Keywords:** attitudes toward competition, perceived competitive environment, student competition, lying flat movement, Goblin Mode, gender differences, PISA 2018

## Abstract

**Introduction:**

In recent years, “lying flat” has emerged as a significant term in contemporary discourse, referring to the phenomenon where modern young people choose passive resistance due to a lack of motivation when facing various situations. These trends have the potential to diminish the future learning enthusiasm and holistic development of vocational high school students, thus impacting their competitiveness in the future job market.

**Methods:**

This study employs a motivational model for vocational high school students based on self-efficacy theory and person-environment fit theory to explore whether the competitive atmosphere moderates the impact of self-efficacy and competitive attitudes on task motivation. Using a secondary data research approach, the study analyzed a sample of 944 Taiwanese vocational high school students from the 2018 Programme for International Student Assessment (PISA) dataset with Partial Least Squares Structural Equation Modeling (PLS-SEM).

**Results:**

The results indicate that self-efficacy and competitive attitudes positively influence task motivation. Additionally, self-efficacy not only enhances competitive attitudes but also indirectly influences task motivation through the mediation of competitive attitudes. The study also revealed that the moderating effect of the competitive environment was not statistically significant in the overall sample. Further multiple-group comparisons between male and female vocational high school students showed no significant differences in most paths, but gender differences emerged in the positive moderating effect of the competitive environment on the influence of competitive attitudes on task motivation.

**Conclusion:**

These findings suggest that in cultivating task motivation among vocational high school students, interventions should be tailored to accommodate the differing responses of male and female students. For female students, providing more opportunities for teamwork and utilizing collaborative approaches to cope with the competitive environment may enhance task motivation. Conversely, for male students, enhancing self-efficacy and stimulating intrinsic competitive attitudes may be more effective. Addressing these differences could potentially reduce the occurrence of the “lying flat” phenomenon among vocational high school students in the future.

## 1 Introduction

Taiwan’s vocational education system plays a crucial role in industrial development, primarily responsible for cultivating technical talent to drive economic growth. It focuses on vocational skills and practical knowledge, equipping students with the necessary abilities for employment (Chen, 2022). However, research shows that vocational high school students generally have lower academic performance and motivation compared to their peers in regular high schools. Nevertheless, when vocational high school students’ intrinsic and extrinsic motivation is stimulated through specific competitions or well-designed learning activities, their academic performance shows little difference from that of regular high school students (Liu, 2020). In other words, enhancing the motivation of vocational high school students is critical for improving their academic performance and fostering their future development.

Since 2021, “lying flat” has become a popular term among young people, symbolizing their passive response to social pressure by reducing their motivation and withdrawing from competition (Yin et al., 2023). “Lying flat” has gradually evolved into a new attitude for coping with external pressures, where young individuals adopt behaviors of “not striving,” “inaction,” and “non-resistance” in response to life’s challenges (Lu et al., 2023). This phenomenon is not limited to Asia. With the slowdown of economic development and the decline in social mobility worldwide, many young people have started to question the value of hard work and effort, as seen in examples like the “Goblin Mode” in the UK and the “Hikikomori” phenomenon in Japan. They actively embrace the “lying flat” attitude, lowering their internal drive and voluntarily withdrawing from various competitions in both life and work (Yin et al., 2023). What appears to be an irresponsible lifestyle is, in fact, closely related to a lack of “motivation” for self-growth (Hsu, 2022; Zheng et al., 2023).

Many theories of motivation have proposed various beliefs related to student learning (Seifert, 2004). Previous studies have found that high levels of job motivation are linked to job performance, productivity, and other related factors. Specifically, individuals with high job motivation voluntarily invest more effort into tasks (Bińczycki et al., 2023; Lohela-Karlsson et al., 2022). Task motivation, particularly in vocational high school students, serves as an internal force that drives individuals to focus on solving challenging problems, mastering skills, or completing tasks (Morgan et al., 1990). Therefore, enhancing task motivation and self-efficacy can be seen as effective strategies to prevent the “lying flat” phenomenon among young people (Cheng and Nguyen, 2022; Jeong et al., 2021). Vocational high school students are currently undergoing a crucial phase of identity transformation, particularly at this stage of their education (Ye et al., 2023b). Given the significant role vocational education plays in both economic and social development, it is essential for these students to enhance their task motivation. By doing so, they can improve academic performance, accumulate necessary skills, and better prepare themselves to meet the demands of the future job market (Chuang et al., 2022; Peng et al., 2022).

According to previous research, self-efficacy is one of the key antecedents of task motivation (Lamb et al., 2019). The relationship between self-efficacy and task motivation has been extensively explored by scholars in the field of education (Schoenherr, 2024; Xie et al., 2024). Bandura’s Self-efficacy Theory posits that in the initiation, enhancement, and maintenance processes of motivation, the most crucial element is an individual’s belief in their own abilities (self-efficacy beliefs). Self-efficacy is the specific manifestation of individuals’ beliefs in their abilities and expectations of success (Bandura, 1978; Bandura, 1986). Students with higher levels of self-efficacy tend to demonstrate more positive task motivation and achieve better academic performance (Chen and Tu, 2021; Pumptow and Brahm, 2020). From this, it can be seen that self-efficacy is one of the most important factors influencing task motivation among vocational high school students.

Competition is an inevitable social context in both learning and work, reflecting individuals’ beliefs about their enjoyment of competition (Wang et al., 2018). Stable personal traits and external environmental factors influence competitive attitudes, with self-efficacy being a key factor (Mildawani et al., 2022). Individuals with a high competitive attitude tend to invest more effort and achieve better performance (Eber et al., 2021; Wang et al., 2018). In vocational high school students, competitive attitudes mediate the relationship between self-efficacy and task motivation. Furthermore, the competitive environment shapes cognition and behavior, often serving as a supportive factor for success (Spurk et al., 2018; Ye et al., 2020). However, the interaction between individual traits and environmental factors is critical, yet understudied, especially in the context of task motivation among vocational high school students. This study explores how the competitive environment moderates the relationship between self-efficacy, competitive attitudes, and task motivation (Rauthmann, 2021).

Since 2021, the concept of “lying flat” has received widespread attention. This term describes the passive coping behavior of young people who, lacking motivation, choose to disengage in response to various societal pressures. Reviewing past studies, it is found that few have introduced the “competitive context” to analyze the relationship between self-efficacy and task motivation among vocational high school students. However, “competition” is an unavoidable social context, and both individual competitive traits and external competitive environments become important factors influencing student task motivation. This study employs self-efficacy theory to understand these phenomena and support youth coping mechanisms. We propose a mediation model where self-efficacy influences task motivation through competitive attitudes. The “person-environment fit” theory is used to examine the competitive environment’s moderating role. This approach, supported by theory and data, allows for an in-depth analysis of self-efficacy and task motivation mechanisms. Our research aims to provide evidence-based recommendations for enhancing task motivation among vocational high school students.

Finally, this study utilizes data from the Programme for International Student Assessment (PISA) 2018, a large-scale international assessment, featuring rigorously designed surveys with strong reliability and validity and focusing on global competitiveness, adaptability, and task motivation among students, which aligns closely with the themes of this study. The dataset includes a diverse cohort of vocational high school students from multiple countries, ensuring representativeness (Chuang et al., 2022). Employing this dataset not only guarantees the reliability of the research but also establishes a foundation for cross-national comparisons. Furthermore, it provides critical insights for the formulation of future educational policies and the enhancement of practice (Anderson et al., 2007; Fischer et al., 2019; Giberti and Maffia, 2020).

## 2 Literature review and research hypotheses

Based on the aforementioned context of vocational high school students dealing with the phenomena of “lying flat” in society, the following literature review and hypothesis derivation are conducted:

### 2.1 The relationship between self-efficacy and motivation to master tasks

**Self-efficacy** refers to the confidence and belief level of individuals in their ability to accomplish specific tasks or achieve particular goals (Bandura, 1997). Individuals with high self-efficacy are more willing to take on challenging tasks and persist in their efforts when faced with difficulties until they achieve their goals (Cattelino et al., 2019). Self-efficacy was proposed by psychologist Bandura, who believed that human behavior is influenced not only by external stimuli and internal drives, but also by individuals’ beliefs in their own abilities (Bandura, 2006). In the framework of self-efficacy theory, self-efficacy plays a key role in people’s behavior of choice, persistence, and completion of specific tasks (Chuang et al., 2022).

**Motivation for the task:** Motivation is the driving force behind behavior, providing an explanation for why individuals act in certain ways. For vocational high school students, motivation is a key determinant of learning success, directly influencing their ability to absorb knowledge during instructional activities (Ye et al., 2022). The task motivation emphasized in the PISA 2018 assessment aligns with this study’s focus, highlighting the internal force that drives individuals to persistently engage with challenging tasks, master skills, and complete goals (Morgan et al., 1990). Task motivation is typically linked to specific goals, tasks, or activities, and learning is the primary task for vocational high school students. Their motivation initiates and sustains their learning behavior and is a critical factor in learning engagement (Howard et al., 2021). Students with high task motivation display greater autonomy, actively setting goals, planning learning paths, and persistently engaging in learning (Chuang et al., 2022). Task motivation influences vocational high school students’ academic and career performance, engagement, and satisfaction. Enhancing task motivation in these students can boost their enthusiasm for learning, encouraging them to invest more time and effort in their studies, continuously improve their professional skills, and lay a solid foundation for future career development (Chuang et al., 2022; Nong et al., 2023). In summary, vocational high school students with high task motivation are more resilient in the face of challenging tasks. Even when confronted with failure or mistakes, they are less likely to give up. Mastery of task motivation is a fundamental cornerstone in personal growth, closely related to both short-term academic achievements and long-term development.

**The relationship between self-efficacy and task motivation:** For vocational high school students, self-efficacy, as an important psychological resource, enhances their task motivation (Bandura, 1997). Bandura’s “Self-Efficacy Theory” is applicable for explaining the relationship between self-efficacy and task motivation in the educational field. When individuals believe they possess the ability and skills required to complete a task (high self-efficacy), they are more motivated to face challenges and willingly invest more time and effort into completing the task (Conradty and Bogner, 2022; Howard et al., 2021). Because of the strong affirmation of their own abilities, individuals can maintain a strong task motivation even in the face of setbacks, believing they can overcome difficulties and achieve their goals (Hong et al., 2021b).

Additionally, “mastery goals” are one of the primary types of academic goals in Achievement Goal Theory. Mastery goals reflect individuals’ desire to develop their abilities through effort and learning (Dweck, 1986). Previous research has found a close relationship between self-efficacy and mastery goals. Individuals with high self-efficacy demonstrate a stronger orientation toward mastery goals in goal setting (Quinonez and Garduño, 2023). “Mastery goal” orientation is typically associated with more proactive learning, deeper learning strategies, and better academic performance (Tuominen et al., 2020). In summary, self-efficacy plays a crucial role in task motivation. For students, self-efficacy serves as an important psychological resource that stimulates high levels of task motivation. In light of this, the following hypothesis was proposed:

H1: Self-efficacy has a positive impact on task motivation.

### 2.2 The effect of competitive attitude as a mediator between self-efficacy and task motivation

Competitive attitude refers to the attitude individuals exhibit in competitive situations, reflecting their preference for competition (Eber et al., 2021). Individuals with a constructive competitive attitude prioritize the development of their overall competence and competitiveness, viewing competition as a catalyst for personal growth. They strive for victory in competition but do not do so at the expense of others (Grasseni and Origo, 2018). A positive competitive attitude is positively correlated with motivation, efficiency, social adaptation, satisfaction, and wellbeing (Barrios, 2014; Ding et al., 2023). In other words, individuals with a high level of competitive attitude believe in their ability to accomplish goals and surpass others. They trust that their overall capabilities are sufficient to support them in achieving goals through effort, thus demonstrating higher task motivation (Toth et al., 2022; Wang et al., 2024). “Considering previous research, this study proposed the following hypothesis:”

H2: Competitive attitude has a positive impact on task motivation.

According to the self-efficacy theory, self-efficacy influences individuals’ behaviors and attitudes, including their competitiveness. Research has found that individuals with high self-efficacy exhibit stronger tendencies toward competitiveness (Mildawani et al., 2022). This situation may involve two motivational mechanisms: Firstly, individuals with high self-efficacy typically possess a more positive psychological state, such as confidence, optimism, and a sense of control. These positive attitudes can enhance a positive competitive attitude (Moke et al., 2018). Secondly, individuals with higher self-efficacy tend to set goals that are challenging and difficult. This goal-setting behavior translates into a competitive attitude, motivating them to persist in the face of difficulties, strive to surpass others, and ultimately achieve their goals (Dissanayake et al., 2019). From this perspective, individuals with high self-efficacy exhibit confidence in their abilities and demonstrate a more positive psychological state and more challenging goal-setting behavior in competitive environments. These factors contribute to a more proactive competitive attitude. Therefore, the following hypothesis was proposed:

H3: Self-efficacy has a positive impact on competitive attitude.

According to the predictions of hypotheses H2, and H3, this study suggests that vocational high school students with high self-efficacy, when perceiving competitive pressure in the learning environment, are more inclined to view competition as an opportunity. Because they are confident in their abilities, they exhibit a more proactive competitive attitude. This proactive competitive attitude further motivates vocational high school students to possess sufficient intrinsic drive when facing difficulties and challenges in learning, thereby enhancing their task motivation. In other words, enhanced self-efficacy among vocational high school students strengthens their competitive attitude, thereby exerting a positive influence on task motivation. From this, the following hypothesis is proposed:

H4: Competitive attitude plays a mediating role in the process, through which self-efficacy influences task motivation.

### 2.3 The moderating effect of competitive environment

Creating an effective educational environment has always been one of the key issues in the field of education (Ye et al., 2023a). The competitive atmosphere within the educational environment can be seen as an abstract environmental variable that influences students’ academic performance and task motivation (Lamb et al., 2019). According to previous research, the competitive environment is a factor that enhances task motivation and promotes performance (Lee et al., 2022). The organizational environments of vocational schools include but are not limited to a competitive atmosphere, a practice-oriented environment, and collaborative and supporting surroundings (Gan, 2023; Monteiro et al., 2021). The competitive environment is one of these commonly used in vocational schools. Vocational schools frequently employ grading and ranking systems, skill competitions, academic competitions, scholarships, and other methods to foster a competitive environment. These strategies aim to raise students’ academic achievement, professional competencies, and motivation for learning (Jaramillo-Mediavilla et al., 2024; Rudolf and Lee, 2023). From this, we assume that students perceive a high level of competition on campus, they often exhibit stronger task motivation, competitive attitudes, and academic engagement.

The competitive environment is one of the significant external factors directly influencing students’ academic performance and career development. According to the “person-environment fit” theory, the interaction between individual traits and environmental factors affects individuals’ cognition, emotions, and behavioral choices (Choy and Yeung, 2023; Rocconi et al., 2020). Based on this, this study infers that there may be an interaction between self-efficacy and the competitive environment. In other words, students with high self-efficacy, when sensing the competitive atmosphere in the learning environment, are more inclined to perceive it as a motivating factor because they believe in their ability to overcome challenges and surpass opponents. Consequently, they are more likely to actively invest additional effort to achieve their goals, demonstrating stronger task motivation.

Furthermore, according to social cognitive theory, students with high self-efficacy believe in their abilities, and they may engage in upward social comparisons to further motivate themselves for improvement (Dissanayake et al., 2019; Esteves et al., 2021). In a competitive environment, high self-efficacy students tend to view academically successful peers as benchmarks. They perceive their excellent performance as motivation rather than a threat when facing peer comparisons. This mindset fosters a willingness to chase or even surpass outstanding peers, leading to a stronger competitive attitude (Mildawani et al., 2022). From this, it can be observed that there may be an interaction between the competitive environment and self-efficacy in influencing the competitive attitude of vocational high school students.

Finally, this study argues that the competitive environment influences individuals’ competitive attitudes. When students perceive a higher level of peer competition in the campus context, they may develop a stronger competitive attitude to enhance their academic competitiveness. This proactive competitive attitude, in turn, stimulates stronger task motivation (Mildawani et al., 2022; Wang et al., 2018).

Based on this, the study posits that the competitive environment moderates the influence of competitive attitude on task motivation among vocational high school students, leading to the following hypotheses:

H5: Competitive environment moderates the relationship between self-efficacy and competitive attitude.

H6: Competitive environment moderates the relationship between self-efficacy and task motivation.

H7: Competitive environment moderates the relationship between competitive attitude and task motivation.

### 2.4 Gender differences in the moderating effect of the competitive environment

The “Person-Environment Fit” theory emphasizes the significant impact of the interaction between personal factors and environmental variables on individual behavior. When there is a good match between an individual’s traits and the environment they are in, it produces positive effects on the individual, such as learning performance, motivation, and career achievements (Bohndick et al., 2022; Choy and Yeung, 2023; Inoue et al., 2021; Koekemoer et al., 2023). Further research has found that the predictive power of the match between individual traits and environmental variables for positive outcomes may be moderated by other influencing factors (Quy and Zhu, 2024; van Zyl et al., 2022). Among these, studies have discovered gender differences in the degree of person-environment fit (Rocconi et al., 2020). In consideration of past research, this article infers that there may be gender differences in the interaction between students’ personal characteristics such as self-efficacy and competitive attitude, and the competitive environment, affecting task motivation. The moderating effect of the competitive environment on the relationship between self-efficacy, competitive attitude, and task motivation may vary by gender. Hence, the following hypothesis was proposed:

H8: There are gender differences in the moderating role of the competitive environment.

The increasing prevalence of the “lying flat” mentality among young people in today’s society is an important phenomenon that warrants our attention (Lu et al., 2023; Zhou, 2023). “Lying flat” implies a lack of drive for self-improvement and is a specific behavior of insufficient motivation (Zheng et al., 2023). This phenomenon has a detrimental impact on students’ academic and personal development. To curb the trend of “lying flat” spreading on campus, in this study, we analyze how personal traits (self-efficacy and competitive attitude) influence motivation, in addition, based on an organizational environment perspective, we also explore the interaction between individual variables and the organizational environment. The organizational environment includes a positive competitive atmosphere, a supportive and caring atmosphere, a teamwork atmosphere, and so on (Lee et al., 2022; Monteiro et al., 2021; Rusticus et al., 2023). Among them, prior research indicates that a competitive environment can boost motivation and encourage academic performance among students. Therefore, in this study, we integrate stable personal traits, like self-efficacy and competitive attitude, with the environmental stimulus of competition to examine an effective way to raise vocational high school students’ task motivation and prevent the spread of the “lying flat” phenomenon.

Drawing on the literature discussed above, the self-efficacy theory constructs a foundational model of the relationship between self-efficacy and competitive attitude on task motivation. From the perspective of person-environment fit theory, the study investigates whether the competitive atmosphere among students will have different effects on the pathways of this model. Finally, differences among vocational high school students of different genders are incorporated into the research model. The aforementioned supporting literature is summarized in Table 1 below.

**TABLE 1:** Research hypotheses of the proposed model.

		Hypothesis	References
H_1_	SE → MT	Self-efficacy has positive effect on task motivation.	Conradty and Bogner, 2022; Hong et al., 2021b; Howard et al., 2021; Quinonez and Garduño, 2023
H_2_	CA → MT	Competitive attitude has positive effect on task motivation.	Toth et al., 2022; Wang et al., 2024
H_3_	SE → CA	Self-efficacy has positive effect on competitive attitude.	Dissanayake et al., 2019; Mildawani et al., 2022; Moke et al., 2018
H_4_	SE → CA → MT	Self-efficacy has an indirect effect on task motivation through competitive attitude	Chen and Tu, 2021; Mildawani et al., 2022; Toth et al., 2022; Wang et al., 2024
H_5_	CE × SE → CA	Competitive environment moderates the effect of self-efficacy on competitive attitude.	Mildawani et al., 2022; Wang et al., 2018
H_6_	CE × SE → MT	Competitive environment moderates the effect of self-efficacy on task motivation.	Chen and Tu, 2021; Gergen et al., 2018; Wang et al., 2017
H_7_	CE × CA → MT	Competitive environment moderates the effect of competitive attitude on task motivation.	Toth et al., 2022
H_8_	Gender	Gender differences will moderate the relationships in H1 to H7	Cheng and Nguyen, 2022; Eber et al., 2021; Fish et al., 2023; Flory et al., 2018; Wang et al., 2018

## 3 Research method

**FIGURE 1:**
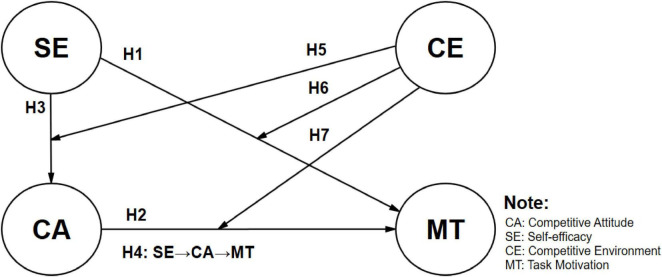
Research model.

The study, based on the literature discussed earlier, aims to investigate the relationship between self-efficacy, competitive attitude, competitive atmosphere, and task motivation among vocational high school students. Drawing from the aforementioned literature, the research framework diagram (see Figure 1 below) is constructed. The study adopts a secondary research method, utilizing the Programme for International Student Assessment (PISA) conducted by the Organization for Economic Co-operation and Development (OECD) every three years. The relevant explanations are as follows:

### 3.1 Research procedure

PISA 2018’s theme focused on students’ global competitiveness, including their adaptability and task motivation, which aligns with the theme of this study. Additionally, PISA questionnaires undergo a rigorous development process, ensuring good reliability and validity. Its use of sampling and survey methods ensures high representativeness of real-world situations (Chuang et al., 2022). Therefore, this study utilized the PISA 2018 data provided on the official PISA website^1^ and selected the necessary data for analysis. For data analysis, this study utilized the Smart PLS 4.1.0.2 software, which employs Partial Least Squares Structural Equation Modeling (PLS-SEM). PLS is suitable for analyzing data with non-normal distributions. It allows for the simultaneous estimation of both mediation and moderation effects within the same model. Moreover, it can estimate complex statistical models with fewer samples (Cheah et al., 2021; Hair et al., 2019; Joreskog, 1982; Sarstedt et al., 2020; Shiau et al., 2019).

This study selected 1,010 original questionnaire responses from Taiwanese vocational high school students from the PISA 2018 data. After removing questionnaires containing missing data and values, a total of 944 valid questionnaires were obtained. Predictive analysis was conducted on this data using Smart PLS. The results of this analysis will be detailed in the subsequent sections.

### 3.2 Measurements

Based on the literature review discussed earlier, this study utilized data from PISA 2018, selecting variables relevant to our research objectives: Self-efficacy, Task Motivation, Competitive Attitude, and Competitive Environment. The following sections describe each variable in detail:

#### 3.2.1 Self-efficacy

Educational scholars often explore self-efficacy, which originates from social cognitive theory. Its primary concept emphasizes individuals’ belief in their ability to accomplish tasks or overcome difficulties (Bandura, 1977). In the vocational domain, self-efficacy refers to vocational high school students’ belief in their confidence level to utilize existing skills to accomplish specific job tasks. Vocational high school students with high self-efficacy beliefs can maintain confidence in their ability to accomplish various tasks or engage in different learning activities, even when facing challenging situations (Chuang et al., 2022; Hong et al., 2021a). In the 2018 PISA survey, self-efficacy measurement included five specific items, such as “feeling proud of one’s accomplishments,” “being able to handle many things at once,” “being able to get through difficult situations on one’s own,” “finding solutions to problems in difficult situations,” and so on. This survey employed a Likert 4-point scale (1 = strongly disagree, 2 = disagree, 3 = agree, 4 = strongly agree). Higher scores on the self-efficacy measurement indicate better self-efficacy among vocational high school students. The Cronbach’s α for self-efficacy in this study was 0.837.

#### 3.2.2 Task motivation

Task motivation is used to denote a key factor influencing vocational high school students, encompassing their willingness to invest in and concentrate on completing specific tasks or learning activities. It includes various facets of motivation such as perseverance, problem-solving, control, and both intrinsic and extrinsic motivation (OECD, 2013; OECD, 2020). Vocational high school students with high motivation levels are likely to possess higher educational and career aspirations and are more inclined to invest in their learning, deriving psychological satisfaction and happiness from the self-growth achieved through this process (Chuang et al., 2022; Michael and Kyriakides, 2023; Ye et al., 2022). In the PISA 2018 dataset, this concept is operationalized under the name “Motivation to master tasks,” which is assessed through questions that measure both work motivation and achievement motivation. This includes evaluating student responses to four statements: “I make an effort to find satisfaction in my work,” “Once I start a task, I always strive to finish it,” “One of the pleasures I get from doing things is when I exceed my past achievements,” and “If it is something I’m not good at, I keep working at it until I master it, rather than switch to something I’m good at.” Responses were measured on a 4-point Likert scale from 1 (strongly disagree) to 4 (strongly agree), where higher scores reflect stronger motivation among vocational high school students. The reliability of this measure in our study was indicated by a Cronbach’s alpha of 0.870.

#### 3.2.3 Competitive attitude

Competitive attitude is used to describe the construct that PISA identifies as “Attitudes toward Competition.” This encompasses not only the individual abilities but also the desire of students to surpass others, thereby motivating them to excel through competitive interactions and exhibit specific behaviors and attitudes (Man et al., 2022; Rudolf and Lee, 2023; Zhou and Wang, 2023). Essentially, “Competitive attitude” reflects an orientation toward enhancing self-worth or personal growth by surpassing others or through comparisons (Menesini et al., 2018). In the PISA 2018 dataset, this construct was operationalized through three survey questions: “I enjoy working in situations where I compete with others,” “It is important for me to perform better than others at work,” and “I try harder when competing with others.” Responses were measured on a 4-point Likert scale ranging from 1 (not at all true) to 4 (extremely true), where higher scores indicate a stronger competitive motivation among vocational high school students. The reliability of this measure, as indicated by a Cronbach’s alpha of 0.776, confirms its robustness in assessing the competitive attitudes among students as conceptualized in this study.

#### 3.2.4 Competitive environment

The competitive environment is an abstract environmental variable that significantly influences students’ learning and academic performance. It is generally understood as a motivational tendency aimed at surpassing others, particularly in contexts where individual success is perceived as contingent upon the failure of others. Students’ perception of the competitive environment primarily reflects their awareness of academic competition and comparison among peers in their educational and daily settings (Rudolf and Lee, 2023; Zhou and Wang, 2023). In contrast, the 2018 PISA survey employed the term “Student Competition” to describe similar phenomena, measuring it by asking students about their perceptions of their school environment. This included four specific questions: “Students seem to value competition,” “There seems to be mutual competition among students,” “Students generally consider mutual competition important,” and “Students feel they are compared to others.” Responses were gathered using a 4-point Likert scale, ranging from 1 (not at all true) to 4 (extremely true). While these responses are subjective, they effectively capture the “school atmosphere,” particularly the competitive atmosphere as perceived by vocational high school students (OECD, 2019; Zhou and Wang, 2023). Hence, what is referred to as “Student Competition” in PISA aligns with what is conceptualized as the “competitive environment” in this study, providing a framework for interpreting how vocational high school students perceive competitive interactions among their peers. Higher scores on the survey indicate a stronger perception of this competitive atmosphere. The reliability of this measure in our study was confirmed by a Cronbach’s alpha of 0.941, illustrating its robustness in assessing the competitive environment within vocational education.

## 4 Results

This study aims to investigate the impact of self-efficacy and competitive attitude on task motivation among vocational high school students in a competitive environment. It adoped a secondary data research method, utilizing data from the Programme for International Student Assessment (PISA) conducted in 2018 for analysis. The results are as follows:

### 4.1 Participants

This study analyzed data from 944 Taiwanese vocational high school students. Regarding school types, among the participants, 458 (48.5%) were from public schools, while 486 (51.5%) were from private schools, indicating a relatively balanced distribution of participants from both types of schools. In terms of school geographical location, participants were mainly distributed in urban areas (450, 47.7%), suburbs (348, 36.9%), and rural areas (146, 15.5%), with the highest proportion of students from urban areas. Regarding gender, there were 535 male participants (56.7%) and 409 female participants (43.3%), indicating a slightly higher proportion of male participants than female participants (Refer to Table 2 for details).

**TABLE 2:** Descriptive analysis (*N* = 944).

Background	*N*	%
**1. School type**		
(1) Public	458	48.5
(2) Private	486	51.5
**2. Gender**		
(1) Female	409	43.3
(2) Male	535	56.7
**3. Areas**		
(1) Urban	450	47.7
(2) Suburban	348	36.9
(3) Rural	146	15.5

### 4.2 Measurement model

This study followed the recommendation of scholars to conduct a complete Structural Equation Modeling (SEM) analysis in two steps. The first stage involves validating the measurement model to ensure that the measurement of each construct meets the recommended standards before proceeding to the second stage, the structural model (Anderson and Gerbing, 1988). In order to ensure rigor, this study conducted a thorough examination of the data before proceeding with the measurement model analysis. It was preliminarily confirmed that there were no missing values in any measurement items, and that the maximum and minimum values of all items fell within the designed range of the scales. After confirming the data integrity, a univariate normality check was conducted. The results of the univariate normality check showed that the skewness values ranged from −0.858 to 0.070, and the excess kurtosis values ranged from −0.487 to 2.430. All values met the recommendations for univariate normal distribution by scholars, where the absolute value of skewness should be less than 2 and the absolute value of excess kurtosis should be less than 7 (Kline, 2005). Based on the above, it is evident that there were no response errors in the analyzed data, and each item conforms to the assumption of univariate normal distribution. Therefore, it is suitable to use the Structural Equation Modeling (SEM) statistical approach for validation.

#### 4.2.1 Convergent validity

In the validation of the measurement model, this study assessed the reliability and validity of each construct by examining multiple items per construct. Parameters such as factor loading (FL), composite reliability (CR), and average variance extracted (AVE) were estimated to evaluate whether each construct possesses adequate reliability and validity. The results are as follows: self-efficacy, consisting of 5 items, the factor loadings range from 0.785 to 0.854; task motivation, comprising 4 items, the factor loadings range from 0.761 to 0.898; competitive attitude, consisting of 3 items, the factor loadings range from 0.811 to 0.876; and for inter-competitive environment, including 4 items, the factor loadings range from 0.915 to 0.947. All factor loadings (FL) for the items are not less than 0.7. In terms of composite reliability, all constructs in this study are greater than 0.7 (0.904, 0.906, 0.883, 0.963). The average variance extracted (AVE) for all constructs are all greater than 0.5 (0.654, 0.707, 0.866, 0.866). From the above, it can be concluded that the measurement of each construct in this study meets the recommendations of scholars, demonstrating good reliability and validity, suitable for further structural model analysis (Chin, 1998; Fornell and Larcker, 1981; Grégoire and Fisher, 2006; Hair et al., 1998) (See details in Table 3).

**TABLE 3:** Construct reliability and validity analysis.

Variable item		FL	CR	AVE
**1. Self-efficacy**				
SE1	I usually manage one way or another.	0.794	0.904	0.654
SE2	I feel proud that I have accomplished things.	0.787		
SE3	I feel that I can handle many things at a time.	0.785		
SE4	My belief in myself gets me through hard times.	0.821		
SE5	When I’m in a difficult situation, I can usually find my way out of it.	0.854		
**2. Task motivation**				
MT1	I find satisfaction in working as hard as I can.	0.842	0.906	0.707
MT2	Once I start a task, I persist until it is finished.	0.898		
MT3	Part of the enjoyment I get from doing things is when I improve on my past performance.	0.761		
MT4	If I am not good at something, I would rather keep struggling to master it than move on to something I may be good at.	0.857		
**3. Competitive attitude**				
CA1	I enjoy working in situations involving competition with others.	0.811	0.883	0.715
CA2	It is important for me to perform better than other people on a task.	0.850		
CA3	I try harder when I’m in competition with other people.	0.876		
**4. Competitive environment**				
CE1	Students seem to value competition.	0.915	0.963	0.866
CE2	It seems that students are competing with each other.	0.947		
CE3	Students seem to share the feeling that competing with each other is important.	0.932		
CE4	Students feel that they are being compared with others.	0.927		

FL, factor loadings; CR, composite reliability; AVE, average variance extracted; SE, self-efficacy; MT, task motivation; CA, competitive attitude; CE, competitive environment.

#### 4.2.2 Discriminant validity

This study utilized both the square root of average variance extracted (AVE) method and the heterotrait-monotrait ratio (HTMT) method to assess discriminant validity between constructs. The results indicated that the square root of AVE for all constructs (e.g., 0.809 for self-efficacy, 0.841 for task motivation, 0.846 for competitive attitude, and 0.930 for competitive environment) is higher than the Pearson correlation values between other constructs, ranging from 0.271 to 0.573 (Fornell and Larcker, 1981). Moreover, all HTMT values between constructs are below the conservative threshold of 0.85 (Henseler et al., 2015). Based on the above, it indicates that the measurement model of this study overall meets the requirements of academic research for discriminant validity, ensuring the independence between different constructs and the overall effectiveness of the model (Refer to Table 4 for details).

**TABLE 4:** Construct discriminate analysis and variance inflation factor.

	AVE	1. SE	2. MT	3. CA	4. CE
1. Self-efficacy	0.654	**0.809**	0.481	0.479	0.327
2. Task motivation	0.707	0.421	**0.841**	0.681	0.340
3. Competitive attitude	0.715	0.402	0.573	**0.846**	0.309
4. Competitive environment	0.866	0.300	0.305	0.271	**0.930**

The figures in bold in the diagonal direction represent the square roots of AVEs; the lower triangular matrix contains the Pearson correlation coefficients; the upper triangular matrix contains the HTMT values.

### 4.3 Structural model

After confirming the convergent validity and discriminant validity of each construct, the overall structural model verification was conducted. In this study, the Partial Least Squares Structural Equation Modeling (PLS-SEM) approach was employed. Regarding model fit, the Standardized Root Mean Square Residual (SRMR) was 0.053, which is smaller than the recommended value of 0.08 by Hu and Bentler (1999). The normed-fit index (NFI) was 0.892, meeting the standard NFI value of 0.8 as suggested by Ullman and Bentler (2001). Lastly, the Goodness of Fit (GOF) index was calculated in this study *GOF* = $\sqrt{\bar{A\,V\,E}\times \bar{R^{2}}}$ = $\sqrt{0.266\times 0.717}$ = 0.436, the Goodness of Fit (GOF) index exceeded the recommended threshold of 0.36 as suggested by Henseler and Sarstedt (2013), Vinzi et al. (2010), and Wetzels et al. (2009). In summary, the model in this study demonstrates strong fit.

#### 4.3.1 Path analysis

**FIGURE 2:**
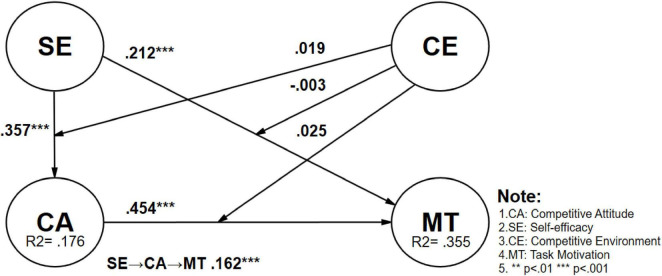
Validation of the research model (complete).

The study treated competitive environment (CE) as a moderating variable, with self-efficacy (SE), competitive attitude (CA), and task motivation (MT) as core variables. Through 10,000 bootstrap iterations, the study tested seven research hypotheses to confirm the relationships among these variables. Firstly, in the analysis of direct effects, it was found that self-efficacy has a significant positive impact on task motivation (H1: SE → MT, β = 0.212, *t*-value = 4.967, *p* < 0.001, CI = [0.129, 0.297]), indicating that students with higher self-efficacy are more motivated to engage in learning activities. Additionally, competitive attitude also has a significant positive impact on task motivation (H2: CA → MT, β = 0.454, *t*-value = 9.982, *p* < 0.001, CI = [0.359, 0.537]), suggesting that students with a more competitive attitude are more easily motivated. In terms of the mediation effects, self-efficacy had a significant direct impact on competitive attitude (H3: SE → CA, β = 0.357, *t*-value = 8.543, *p* < 0.001, CI = [0.271, 0.436]). Through the mediating effect of competitive attitude, self-efficacy indirectly influences task motivation by enhancing competitive attitude (H4: SE → CA → MT, β = 0.162, *t*-value = 6.072, *p* < 0.001, CI = [0.110, 0.215]). This indicates that students with higher self-efficacy not only have a more positive competitive attitude but also indirectly foster better task motivation through their higher competitive attitudes. In summary, hypotheses H1, H2, H3, and H4 are all supported, demonstrating the significant roles of self-efficacy and competitive attitude in the formation of student task motivation. Additionally, concerning the explanatory power of endogenous latent variables, scholars suggest that an explanatory power greater than 0.67 indicates high explanatory power, around 0.33 indicates moderate explanatory power, and less than around 0.19 indicates weaker explanatory power. In this study, the explanatory power of task motivation is 0.355, indicating moderate to high explanatory power, while the explanatory power of competitive attitude is 0.176, which is very close to the suggested weaker explanatory power of 0.19 by scholars. In other words, this study’s overall model demonstrates predictive power both statistically and practically (See Table 5 for details and Figure 2).

**TABLE 5:** Research hypothesis verification.

Hypotheses		Point estimate				Bias-corrected CI 95%		Result
**Variable relationship**		**path coefficient**	**Standard Deviation**	***T* value**	***p*-value**	**2.5%**	**97.5%**	
H_1_	SE → MT	0.212	0.043	4.967	0.000***	0.129	0.297	Accept
H_2_	CA → MT	0.454	0.045	9.982	0.000***	0.359	0.537	Accept
H_3_	SE → CA	0.357	0.042	8.543	0.000***	0.271	0.436	Accept
H_4_	SE → CA → MT	0.162	0.027	6.072	0.000***	0.110	0.215	Accept
H_5_	CE × SE → CA	0.019	0.036	0.537	0.591	−0.052	0.088	False
H_6_	CE × SE → MT	−0.003	0.035	0.072	0.943	−0.074	0.064	False
H_7_	CE × CA → MT	0.025	0.043	0.585	0.559	−0.059	0.112	False

SE, self-efficacy; MT, task motivation; CA, competitive attitude; CE, competitive environment.

***p < 0.001. Bootstrap 10,000 times.

#### 4.3.2 Moderate effect: competitive environment

In the moderating effect analysis, this study examined the moderating effects of competitive environment on the relationships between self-efficacy and competitive attitude, self-efficacy, and task motivation. However, the results indicated that these moderation effects are not significant (H5: CE × SE → CA β = 0.019, *t*-value = 0.537, *p* = 0.591, CI = [−0.052, 0.088]; H6: CE × SE → MT β = −0.003, *t*-value = 0.072, *p* = 0.943, CI = [−0.074, 0.064]; H7: CE × CA → MT β = 0.025, *t*-value = 0.585, *p* = 0.559, CI = [−0.059, 0.112]). This suggests that the level of competition among students does not significantly moderate the relationship between self-efficacy and task motivation or competitive attitude. In summary, hypotheses H5, H6, and H7 are not supported among all vocational high school students, indicating limited moderating effects of competitive environment in the educational context (See Table 5 for details and Figures 2, 5).

#### 4.3.3 Moderate effect: gender

**FIGURE 3:**
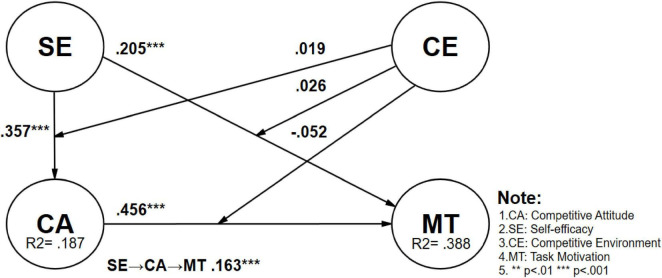
Validation of the research model (male).

**FIGURE 4:**
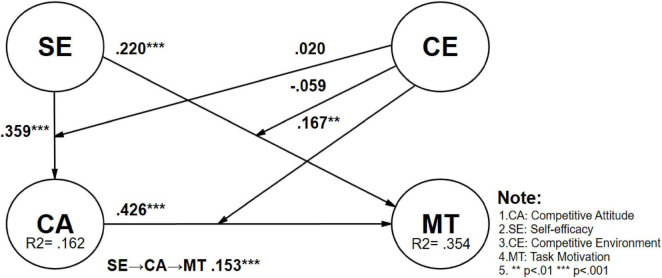
Validation of the research model (female).

**FIGURE 5:**
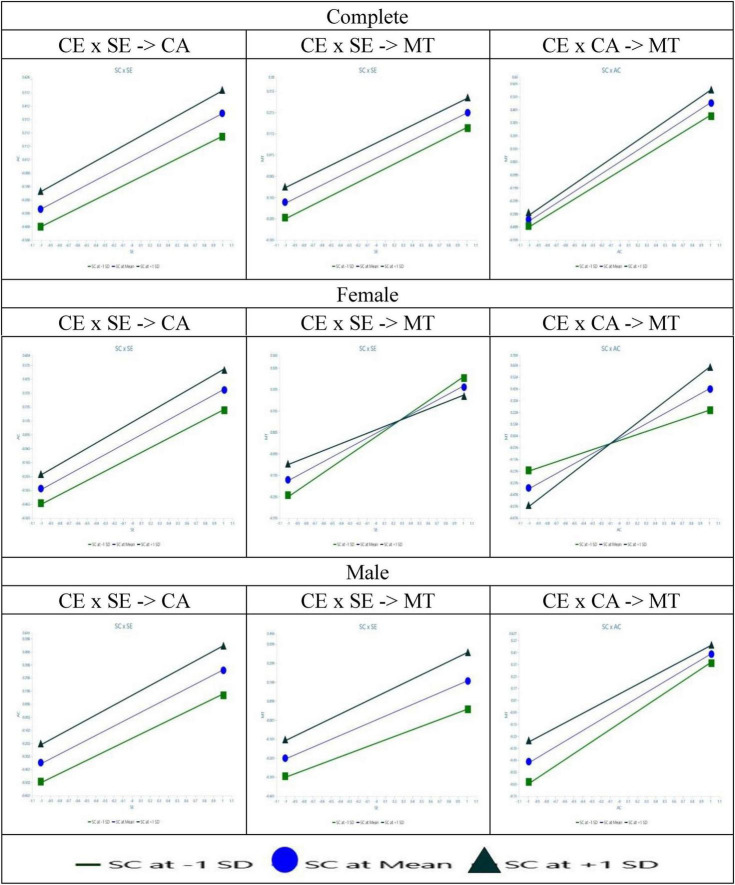
The moderating effects of gender on group differences. SE, self-efficacy; MT, task motivation; CA, competitive attitude; CE, competitive environment.

This study conducted a multi-group analysis on male (*n* = 535) and female (*n* = 409) students to investigate the influence of gender differences among vocational high school students on the research model. After analyzing the six validated paths, the *p*-values for gender differences between male and female students ranged from 0.551 to 0.999, indicating that gender differences on these paths were not significant. However, when exploring hypothesis 7, which examines the moderating effect of competitive environment on the relationship between competition attitude and task motivation (CE x CA → MT), gender differences reached a significant level. For female students, peer competition significantly positively moderated the impact of competition attitude on task motivation (Female: CE × CA → MT β = 0.167, *t*-value = 3.041, *p* = 0.002 < 0.01), whereas this moderating effect was not significant for male students (Male: CE × CA → MT β = −0.052, *t*-value = 0.963, *p* = 0.333). The gender difference was statistically significant (Diff.: β = −0.219, *t*-value = 2.854, *p* = 0.004 < 0.01), indicating that in the vocational education environment, female students’ perception of peer competition significantly enhances the positive impact of competition attitude on their task motivation, while male students’ task motivation is not influenced by the competitive environment (See Table 6 for details and Figures 3–5).

**TABLE 6:** Path coefficients and group difference across gender (male *n* = 535, female *n* = 409).

Variable relationship	Gender	Path coefficient	Standard deviation	*T* value	*p*-value	Difference	*T* value	*p*-value
SE → MT	Male	0.205	0.057	3.598	***	−0.015	0.191	0.849
	Female	0.220	0.057	3.852	***			
CA → MT	Male	0.456	0.061	7.528	***	0.030	0.350	0.727
	Female	0.426	0.062	6.917	***			
SE → CA	Male	0.357	0.057	6.308	***	−0.002	0.021	0.983
	Female	0.359	0.053	6.748	***			
SE → CA → MT	Male	0.163	0.036	4.510	***	0.010	0.199	0.842
	Female	0.153	0.036	4.299	***			
CE × SE → CA	Male	0.019	0.047	0.417	0.677	0.000	0.001	0.999
	Female	0.020	0.053	0.367	0.714			
CE × SE → MT	Male	0.026	0.044	0.596	0.551	0.085	1.255	0.210
	Female	−0.059	0.052	1.140	0.254			
CE × CA → MT	Male	−0.052	0.054	0.969	0.333	−0.219	2.856	0.004**
	Female	0.167	0.055	3.041	0.002**			

SE, self-efficacy; MT, task motivation; CA, competitive attitude; CE, competitive environment.

**p < 0.01;

***p < 0.001.

### 4.4 Discussion

Firstly, this study found that vocational high school students with higher self-efficacy tend to have higher levels of task motivation. This result supports self-efficacy theory, which posits that an individual’s belief in their ability to accomplish specific tasks is central to the initiation, enhancement, and maintenance of task motivation processes (Bandura, 1978; Bandura, 1986). Therefore, vocational high school students with higher self-efficacy are more willing to invest time and effort in completing tasks when faced with challenges (Chuang et al., 2022; Conradty and Bogner, 2022; Howard et al., 2021). In other words, self-efficacy indeed plays a crucial role in enhancing task motivation among vocational high school students.

Secondly, this study found that the competitive attitude of vocational high school students does indeed enhance task motivation. Competitive attitude reflects a desire among vocational high school students to outperform others or to compare themselves with others in order to enhance their sense of self-worth (Bandura, 1978; Bandura, 1986). Previous research has shown that vocational high school students with a healthy competitive attitude perceive competition as a factor that promotes self-growth and focus their attention on developing their own abilities. Therefore, vocational high school students with a high level of competitive attitude are indeed able to demonstrate higher task motivation (Toth et al., 2022; Wang et al., 2024).

Furthermore, this study found that not only vocational high school students with high self-efficacy tend to have higher competitive attitudes, but also competitive attitudes mediate the relationship between self-efficacy and task motivation. Based on this finding, it is evident that vocational high school students with high self-efficacy indeed perceive competition as an opportunity for self-improvement and exhibit more positive competitive attitudes. In a benign competitive environment, this stimulates individuals to exert more effort, thereby enhancing task motivation (Dissanayake et al., 2019; Eber et al., 2021; Mildawani et al., 2022). In other words, vocational high school students’ belief in their ability to complete specific tasks, which is also self-efficacy, not only influences their willingness to face challenges but also affects their task motivation to continue putting in effort.

Moreover, the competitive environment didn’t moderate the relationship between self-efficacy, competitive attitudes, and task motivation. This result contradicts past research suggesting that a competitive environment can motivate individuals to achieve career success or better job performance (Spurk et al., 2018; Ye et al., 2020). Past scholars have argued that human behavior arises from the interplay of personal factors, environmental influences, and purposeful actions (Bandura, 2001; Bandura et al., 2001). Therefore, the facilitating effect of the competitive environment may not be directly regulated by the external environment itself but rather relies on individuals’ cognitive interpretations of the perceived external environmental atmosphere and its impact on internal traits (such as self-efficacy or competitive attitudes). This may also reflect the attitudes of the current “lying flat” generation in response to various pressures in life, exhibiting attitudes of “not striving,” “inaction,” or “non-resistance” (Lu et al., 2023).

Finally, this study further explored the influence of gender differences on vocational high school students in the proposed model. The results showed that the majority of the path analyses did not demonstrate statistically significant differences between genders. The only difference observed was in the moderating effect of the competitive environment on the relationship between competitive attitudes and task motivation, specifically, in female vocational high school students. In this group, the competitive environment positively moderated the impact of competitive attitudes on task motivation, while this moderating effect was not statistically significant among male students. This difference may stem from the different socialization processes and gender role expectations that males and females experience during their upbringing, leading to variations in their responses to pressure and competition. In contrast, female vocational high school students may exhibit relatively weaker competitiveness, but they tend to be more sensitive to changes in their environment. Especially when facing competitive challenges, female students may tend to seek external support and invest more time in learning, whereas male students may not possess these traits as much. Therefore, when female vocational high school students perceive the pressure of a competitive environment, their positive competitive attitude may be more likely to be stimulated, thereby positively influencing their task motivation. In contrast, male students may be less affected by external competition because they may be more accustomed to competitive environments.

## 5 Conclusion

In recent years, the concepts of “lying flat” have sparked intense global debate due to widespread dissemination through the internet. Although this phenomenon has long existed, it now highlights the passive and negative coping mechanisms of modern young people when faced with societal expectations and competitive pressures. To gain a deeper understanding of these phenomena, this study employed a secondary data analysis approach, integrating self-efficacy theory and person-environment fit theory, and conducted an empirical analysis using data from the 2018 Programme for International Student Assessment (PISA). The study’s main findings confirm the self-efficacy theory: vocational high school students’ self-efficacy and competitive attitudes significantly positively affect their task motivation. Insights from the person-environment fit theory reveal a new perspective: the competitive environment enhanced the positive impact of competitive attitudes on task motivation among female students but not among male students. These findings provide new insights into the behavioral patterns of vocational high school students within competitive environments and offer valuable references for further research and the development of educational policies. The following sections will elaborate on the deeper implications and potential impacts of these findings.

### 5.1 Theoretical implications

According to self-efficacy theory, individuals with high self-efficacy exhibit more positive attitudes and tend to set challenging goals (Bandura, 1978, 1986, 1997). This study confirms that self-efficacy and competitive attitudes play crucial roles in enhancing task motivation among vocational high school students. From the perspective of person-environment fit theory, we found significant differences in how males and females perceive and respond to competitive environments. However, the results indicate that the moderating effect of the competitive environment on the relationships between self-efficacy, competitive attitudes, and task motivation was not statistically significant. This finding challenges the current view of person-environment fit theory, which posits that a mismatch between individual traits and work environment characteristics can lead to negative outcomes (Caplan and Van Harrison, 1993). In the contemporary sociocultural context, whether considering the “Lying Flat” phenomenon discussed in this study, the “Goblin Mode” in the West, or Japan’s “Hikikomori,” it is evident that modern vocational high school students may no longer view competition as the primary means of enhancing self-worth. Instead, they increasingly seek development paths that according to their personal goals and interests. Our study targeted Generation Z, born after 2000, and the results show that their response to competitive organizational environments differs from that of previous generations (e.g., Millennials, Generation X) (Inoue et al., 2021; Stephan et al., 2023). The desensitization of Generation Z to competitive organizational environments may be related to their unique upbringing and personal traits, such as diverse value orientations, flexible career attitudes, and varied educational and family backgrounds (Dokoupilová et al., 2024; Talmon, 2019). This study not only highlights the importance of considering individual needs and environmental factors in vocational high school education but also deepens our understanding of the factors influencing task motivation among vocational high school students. We suggest that future research, based on the “person-environment fit” theory, should further refine the characteristics of Generation Z students and explore more diverse organizational atmospheres, such as supportive and caring environments, and collaborative and team-oriented environments (Monteiro et al., 2021). A multilevel model could also be employed to explore in greater detail the differences between external environments and individuals, which would help create an optimal educational environment tailored to the unique characteristics and developmental needs of different students.

### 5.2 Practical implications

The trend of “lying flat” phenomena is closely associated with the lack of motivation among young people. Therefore, enhancing task motivation among vocational high school students can be seen as an effective strategy to reduce the “lying flat” phenomenon. This study demonstrates that enhancing task motivation can be achieved by cultivating self-efficacy and increasing competitive attitude among vocational high school students. Especially in dealing with the competitive environment faced by vocational high school students, male and female responses differ. For female vocational high school students, coping with the competitive atmosphere through teamwork may effectively enhance their task motivation. Conversely, direct external competition may not be as suitable for male vocational high school students. Instead, enhancing self-efficacy and stimulating intrinsic competitive attitudes may be the best approach to improving their task motivation, with the expectation of reducing the occurrence of “lying flat” phenomena among vocational high school students in the future.

### 5.3 Limitations and future study

This study combined the self-efficacy theory and the person-environment fit theory, obtaining preliminary results from the secondary data of PISA 2018. However, there are some limitations to this study. Firstly, since the sampling subjects of this study were vocational high school students aged 15 to 16, the inferential generalization to students of different age groups may be limited. Secondly, considering that the data source is from 2018, although the survey sample is somewhat representative, the inferences from this study may be constrained when applied to the post-pandemic era. Furthermore, considering that different countries have diverse cultural backgrounds, values, and social environments, the applicability of the results of this study may face challenges when conducting cross-national comparisons. Lastly, although the study focuses on the task motivation of vocational high school students, it is important to recognize that individual motivation is profoundly influenced by specific factors such as the environment and the context of individuals, events, times, places, and objects. Given the aforementioned limitations, future research could build upon this study by expanding to different age groups, educational systems, cultural backgrounds, and generations, and further deepen the understanding of the diversity of motivation among vocational high school students.

## Data Availability

Publicly available datasets were analyzed in this study. These data can be found here: https://www.oecd.org/pisa/data/2018database.

## Footnotes

1
https://www.oecd.org/pisa/data/2018database/
